# Dataset on the effect of receiver size and acceptance half-angle on the aperture width and height of compound parabolic concentrator for low-concentrating photovoltaic applications using RSM

**DOI:** 10.1016/j.dib.2021.107630

**Published:** 2021-11-23

**Authors:** Faisal Masood, Nursyarizal Bin Mohd Nor, Perumal Nallagownden, Irraivan Elamvazuthi, Mohammad Azad Alam, Mohammad Yusuf, Mujahid Ali, Javed Akhter, Mubbashar Mehmood

**Affiliations:** a Department of Electrical and Electronics Engineering, Universiti Teknologi PETRONAS, Bandar Seri Iskandar, Perak 32610, Malaysia; b Department of Electrical Engineering, University of Engineering and Technology Taxila, Rawalpindi 47080, Pakistan; c Department of Mechanical Engineering, Universiti Teknologi PETRONAS, Bandar Seri Iskandar, Perak 32610, Malaysia; d Department of Chemical Engineering, Universiti Teknologi PETRONAS, Bandar Seri Iskandar, Perak 32610, Malaysia; e Department of Civil and Environmental Engineering, Universiti Teknologi PETRONAS, Bandar Seri Iskandar, Perak 32610, Malaysia; f Department of Mechanical Engineering, University of Engineering and Technology Taxila, Rawalpindi 47080, Pakistan; g School of Engineering and Physical Sciences, Heriot-Watt University, Edinburgh EH14 4AS, UK

**Keywords:** Concentrating photovoltaics, Concentration ratio, Design optimization, Analysis of variance

## Abstract

The combined effect of design control factors on the response variables gives valuable information for geometric design optimization of the compound parabolic concentrator. This study presents the data related to the statistical modeling and analysis of variance for aperture width and height of a low concentration symmetric compound parabolic concentrator designed for photovoltaic applications. The design matrix was generated using the response surface modeling approach. The geometric design equations of the proposed concentrator were developed and solved analytically using MATLAB. The empirical models were developed to establish relationships between the control factors and response variables of the proposed system. The analysis of variance was conducted for two significant response variables. The developed statistical models can be used to predict the selected response variables within the permissible range. The presented data can be used for statistical modeling and design optimization of the two-dimensional symmetric compound parabolic concentrator.

**Specifications Table**

**Table:** 

Subject	Renewable Energy
Specific subject area	Solar Energy, Low concentration PV systems
Type of data	Tables, Figures, Graphs
How data were acquired	Analytical modeling and simulation using MATLAB/Statistical modeling using Design-Expert software
Data format	Raw, Analysed
Parameters for data collection	Aperture width (Wapr) and height (H) of low concentration compound parabolic concentrator (CPC)-based photovoltaic system.
Description of data collection	The receiver width (Wrec) and acceptance half-angle (ϴa) were varied over a specified range, and the corresponding values of the aperture width (Wapr) and CPC height (H) were determined using MATLAB. In addition, the synergistic impact of control factors on the design responses was analyzed using the response surface modeling approach based on face-centered central composite design.
Data source location	Universiti Teknologi Petronas, Seri Iskandar 32610, Perak, Malaysia. Latitude and longitude for collected data: 4.3838° N, 100.9709° E
Data accessibility	The data are with this article. The source code can be found at the following URL. https://doi.org/10.5281/zenodo.5574777
Related research article	Masood, F., et al., A New Approach for Design Optimization and Parametric Analysis of Symmetric Compound Parabolic Concentrator for Photovoltaic Applications. Sustainability, 2021. 13(9): p. 4606. doi: 10.3390/su13094606.

**Value of the Data**

• The data presented can be used to give an insight into the combined impact of independent design parameters on the significant design responses of a CPC-based low concentration PV system.
• The impact of independent design parameters on the geometry of symmetric 2D Compound Parabolic Concentrator can be grasped from the dataset.
• The researchers working on the design optimization of low concentrating PV systems can get benefit from the given data.
• The optimized design parameters selected from the statistical analysis can be used for fabricating a physical model of the proposed compound parabolic concentrator for experimental investigation.

## 1 Data Description

The dataset presented in this article is related to the computed results reported in the article entitled “A New Approach for Design Optimization and Parametric Analysis of Symmetric Compound Parabolic Concentrator for Photovoltaic Applications” [1]. Solar cells are the major contributors to the net cost of solar PV systems due to costly silicon-based solar cell materials. However, the integration of optical concentrators with solar PV modules results in concentrated solar radiation on the surface of PV cells. Due to concentrated solar radiation, the solar cell material required to generate a given amount of power can be reduced [2]. The costly solar cells can thus be potentially replaced by relatively cheap optical concentrators. The electrical power generated by a concentrated PV system increases by a factor equal to the concentration ratio of its concentrator [3]. A compound parabolic concentrator (CPC) is a non-imaging device generally used in PV, thermal, or hybrid PV/thermal systems as an optical concentrating element [4]. The CPC has been regarded as the best static low-concentrating system for PV applications for the past five decades due to the various advantages like higher optical efficiency and being able to collect both diffuse and direct radiations [5].

**Fig. 1:**
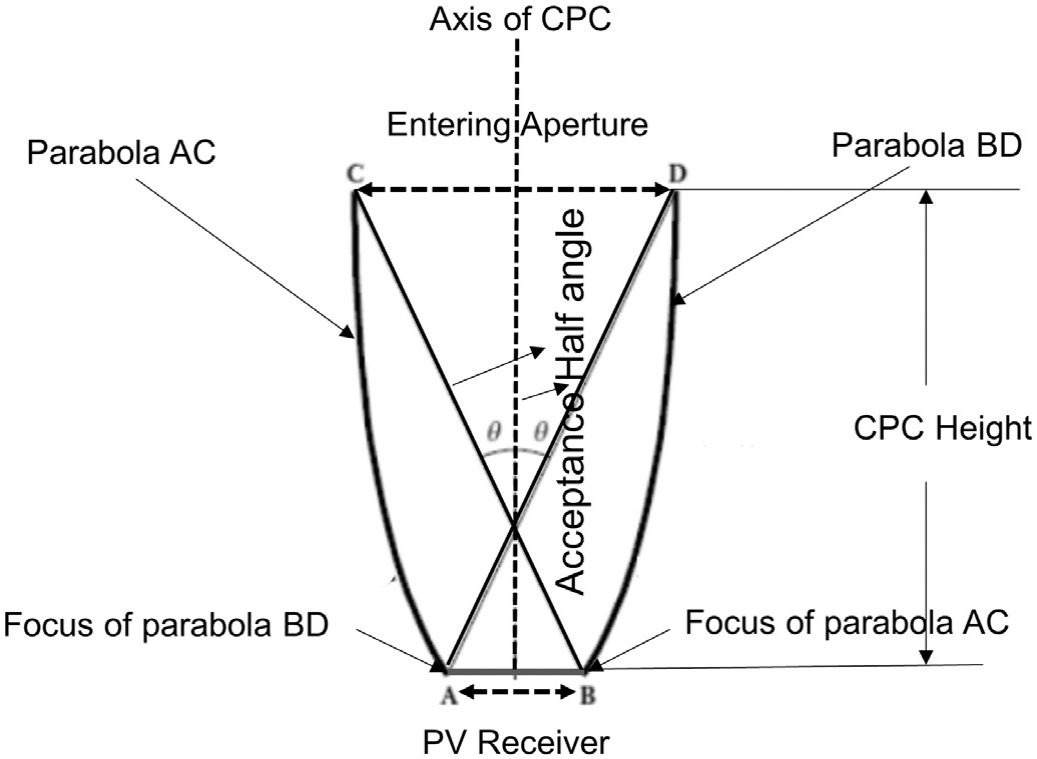
Schematic cross-sectional view of 2D CPC [1].

The schematic diagram of CPC for PV applications is shown in Fig. 1. The design process is required to ensure effective concentration of solar radiation as well as uniform distribution of solar flux on the PV receiver. From the design point of view, the width of the PV receiver and acceptance half-angle are specified as the independent design parameters, whereas the concentration ratio, CPC height, and width of entry aperture are the design responses. The response surface modeling (RSM) approach can be used to develop appropriate approximating empirical models describing the relation between the independent design parameters and the corresponding response variables of a system [6]. The developed empirical models can then be used for the prediction of responses with adequate accuracy.

**Fig. 2:**
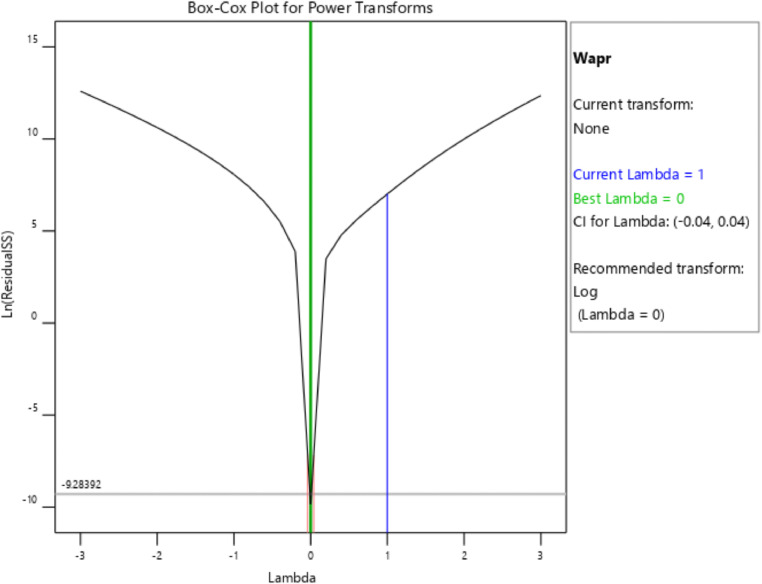
Box-Cox plot for power transforms of *Wapr.*

**Fig. 3:**
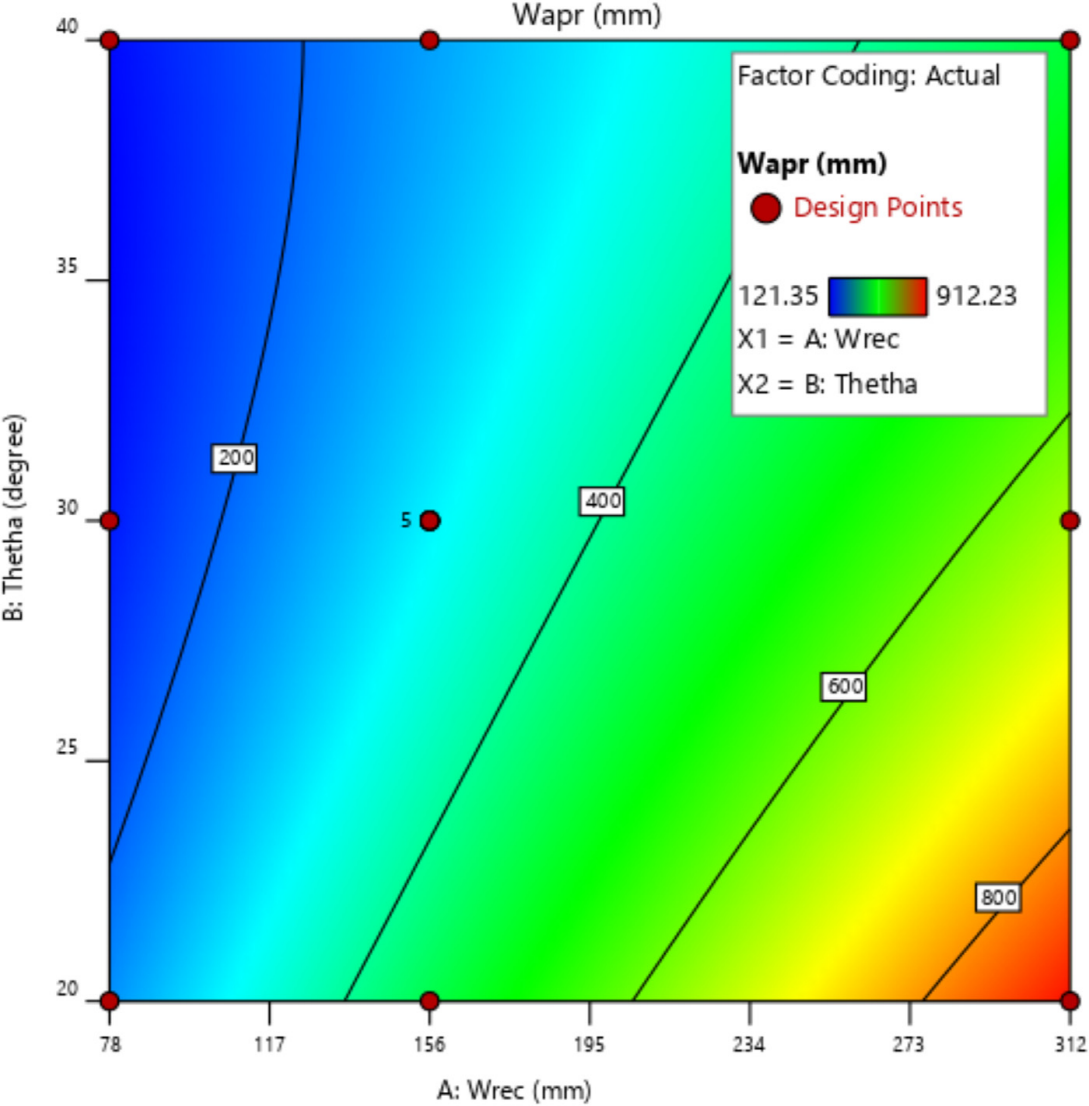
Contour plot showing the effects of independent design parameters on *Wapr.*

**Fig. 4:**
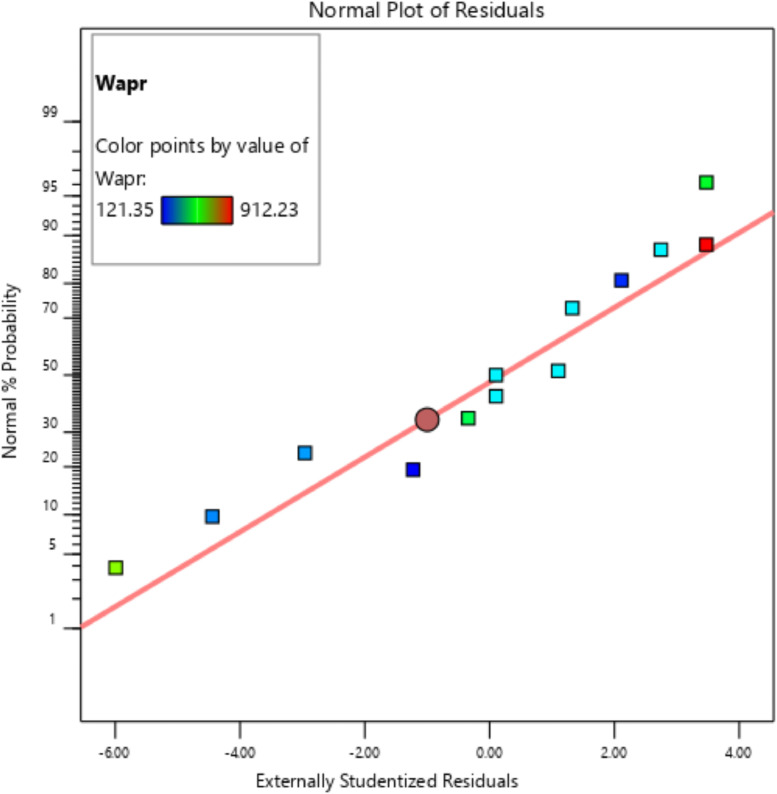
Normal plot of residuals for *Wapr.*

**Fig. 5:**
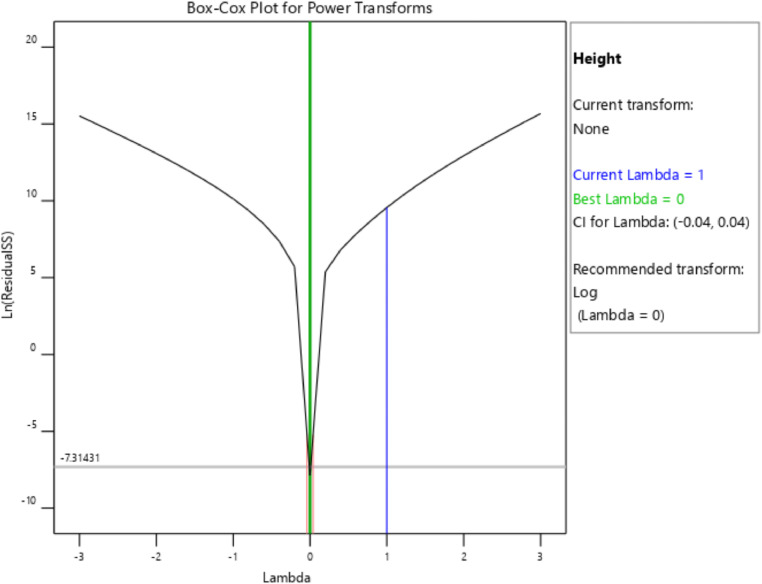
Box-Cox plot for power transforms of CPC height *H.*

**Fig. 6:**
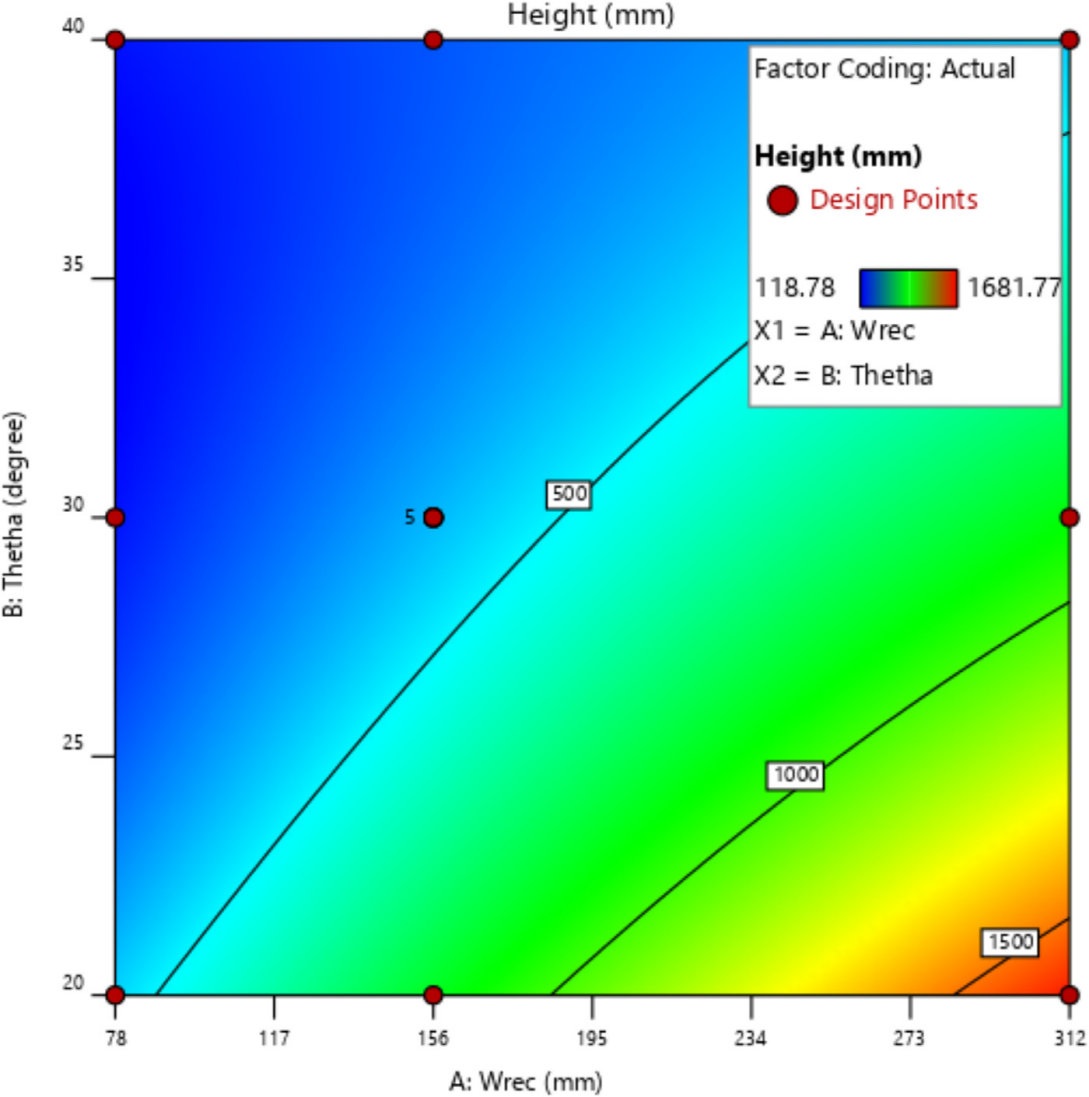
Contour plot showing the effect of design parameters on CPC height *H.*

**Fig. 7:**
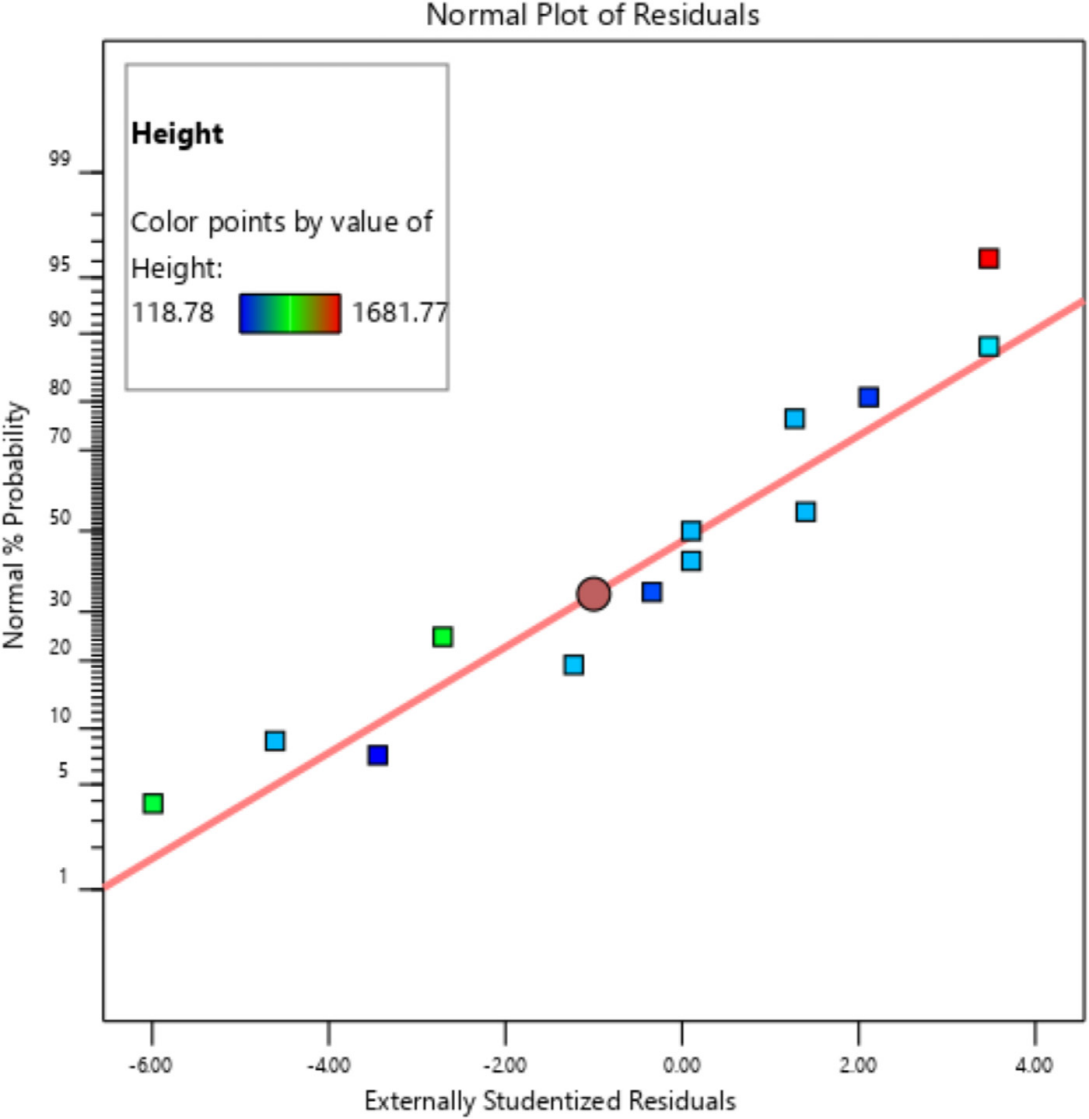
Normal plot of residuals for CPC height *H.*

Table 1 presents the actual and coded values as well as the permissible range for each independent design parameter. The response variables were then specified, and the design matrix was generated based on the central composite design. Table 2 gives the actual design matrix consisting of various combinations of independent design parameters for a total of 13 runs. The corresponding response variables were calculated by simulating the analytical model. The quadratic models were selected for both response variables. The analysis of variance results for the response variables ‛*Wapr*' and CPC height ‛*H*' are presented in Tables 3 and 4, respectively. The quadratic models for the above-mentioned response variables are given in Eqs. (1) and (2). The box-cox plot for power transforms in the case of ‛*Wapr*' is shown in Fig. 2. The combined impact of design control factors on the first response variable ‛*Wapr*' is depicted by the contour plot shown in Fig. 3. The normal plot of residuals for ‛*Wapr*' is shown in Fig. 4. The box-cox plot, contour plot, and normal plot of residuals for the second response variable CPC height ‛*H*' are shown in Figs. 5, 6, and 7, respectively.

**Table 1:** Independent design parameters with ranges and levels.

			Ranges and levels		
Factor	Name	Units	−1	0	+1
A	Receiver Width	(mm)	78	156	312
B	Acceptance Half-angle	(^o^)	20	30	40

**Table 2:** Design matrix and responses.

Experimental Run	Wrec (mm)	Acceptance half-angle (°)	Concentration ratio	Wapr (mm)	CPC Height (H) (mm)
1	0	0	2.00	312.00	405.30
2	0	−1	2.92	456.11	840.88
3	0	0	2.00	312.00	405.30
4	+1	0	2.00	624.00	810.60
5	−1	−1	2.92	228.06	420.44
6	0	0	2.00	312.00	405.30
7	+1	+1	1.56	485.38	475.14
8	0	0	2.00	312.00	405.30
9	0	0	2.00	312.00	405.30
10	0	+1	1.56	242.69	237.57
11	−1	0	2.00	156.00	202.65
12	+1	−1	2.92	912.23	1681.77
13	−1	+1	1.56	121.35	118.78

**Table 3:** ANOVA results for *Wapr.*

Source	Sum of squares	df	Mean squares	*F*-Value	*P*-Value	
Model	5.389E+05	5	1.078E+05	679.97	<0.0001	significant
A-Wrec	3.831E+05	1	3.831E+05	2417.23	<0.0001	
B-Theta	1.049E+05	1	1.049E+05	661.68	<0.0001	
AB	26569.89	1	26569.89	167.63	<0.0001	
A²	7.77	1	7.77	0.0490	0.8311	
B²	4852.85	1	4852.85	30.62	0.0009	
Residual	1109.55	7	158.51			
Lack of Fit	1109.55	3	369.85			
Pure Error	0.0000	4	0.0000			
Cor Total	5.400E+05	12

**Table 4:** ANOVA results for CPC height (H).

Source	Sum of squares	df	Mean squares	*F*-Value	*P*-Value	
Model	1.942E+06	5	3.884E+05	191.10	<0.0001	significant
A-Wrec	8.256E+05	1	8.256E+05	406.24	<0.0001	
B-Theta	8.381E+05	1	8.381E+05	412.41	<0.0001	
AB	2.123E+05	1	2.123E+05	104.48	<0.0001	
A^2^	99.63	1	99.63	0.0490	0.8311	
B^2^	62218.90	1	62218.90	30.62	0.0009	
Residual	14225.68	7	2032.24			
Lack of Fit	14225.68	3	4741.89			
Pure Error	0.0000	4	0.0000			
Cor Total	1.956E+06	12

The quadratic model developed to describe the relationship between the response variable ‛*Wapr*' and the independent design parameters, ‛*Wrec'* and ‛*ϴa'*, is given by Eq. (1), where *A* and *B* represent ‛*Wrec'* and ‛*ϴa',* respectively.

(1) $$Wapr\;=\;395.16\;+\;252.7A\;-\;133.39B\;-\;80.03AB\;-\;1.93A^{2}\;-\;41.92B^{2}$$

The analysis of variance (ANOVA) was conducted to examine the significance of all independent parameters and the model itself. The input is considered to be significant if the *P*-value corresponding to the F-value is less than 0.05.

The Box-Cox plot for power transforms of the response variable ‛*Wapr'* is shown in Fig. 2. The Box-Cox transformation is used to develop uniformity of dispersion. The power transform creates a monotonic transmutation of data via power functions. The best value of lambda (λ=0), in this case, suggests a logarithmic transformation.

The prediction of the response variable ‛*Wapr'* as a function of control factors is shown in Fig. 3. The input parameter ‛*Wrec'* has a more significant impact on the response variable ‛*Wapr'* as compared to ‛*ϴa'.*

The quadratic model for the response variable CPC height ‛*H'* as a function of control factors is described by Eq. (2), where *A* and *B* represent ‛*Wrec'* and ‛*ϴa'* respectively.

(2) $$H\;=\;525.10\;+\;370.94A\;-\;377.07B\;-\;226.24AB\;-\;6.93A^{2}\;+\;150.09B^{2}$$

The developed model was found to be significant from the ANOVA analysis, as shown in Table 4, as the *P*-value is less than 0.0001.

The best value of lambda (λ=0) suggests a logarithmic transformation in this case for the response variable CPC height ‛*H'*.

The contour plot for the response variable CPC height ‛*H'* indicates that the control factor ‛*Wrec'* has a more profound impact on the given response variable than ‛*ϴa'.*

## 2 Experimental Design, Materials and Methods

The design process of non-imaging 2D CPC for PV applications starts with selecting the appropriate values of input geometric design parameters, including the width of the receiver (*Wrec*) and the acceptance half-angle (*ϴa*) [7]. The choice of ‛*Wrec*' and ‛*ϴa*' depends on the output power requirement and the location's geographical coordinates where the CPC-based PV system needs to be installed for stationary or quasi-stationary applications [8]. In the present research, after selecting geometric design factors, the mathematical equations relating the design factors and responses for the desired geometrical shape were developed and solved analytically to produce the values of output design parameters [9]. The complete set of design equations can be found in Ref. [1]. A parametric analysis was then performed to analyze the impact of input design parameters on the output parameters. Finally, a suitable range of values for both design parameters was selected based on the parametric analysis.

For statistical modeling and subsequent geometric design optimization of the proposed CPC-PV collector, Design-Expert 12 software was employed [6]. The RSM tool of Design-Expert software was utilized to generate the design matrix and perform the statistical analysis of the significant geometric design parameters of 2D CPC for PV applications. The ranges and levels of independent design parameters were specified. The central composite design (CCD) approach was used for the model development in RSM to examine the combined impact of independent design parameters on the response variables. The individual and combined interaction effects of control design factors on two crucial design responses, i.e., the width of entry aperture ‛*Wapr'* and CPC height ‛*H'*, were evaluated. The objective of applying CCD to the design parameters used in the present work is to develop the second-order model efficiently.

The analytical model based on the design equations of CPC with a flat receiver surface was simulated using MATLAB as per the design matrix generated by following the CCD approach of RSM. The corresponding values of response variables were determined and substituted in the design matrix, as shown in Table 2. The appropriate quadratic model was selected, and the resulting equations for both responses were found from the model results. The steps involved in the initial geometric design, statistical modeling/analysis, and geometric design optimization process are summarized below:

❖ Select the values of input design parameters.
❖ Develop a mathematical model of 2D CPC geometry for PV applications.
❖ Develop MATLAB code for solving geometric design equations.
❖ Simulate MATLAB model for generating 2D CPC geometry.
❖ Perform parametric analysis using all possible values of input design parameters.
❖ Select a suitable range of values for input design parameters.
❖ Perform statistical analysis and modeling using Design-Expert software.
❖ Perform geometric design optimization as per given constraints for design factors.

The steps mentioned above can be followed for statistical modeling and design optimization of 2D CPC for PV applications, as presented in Ref. [1].

## CRediT authorship contribution statement

**Faisal Masood:** Conceptualization, Methodology, Software, Writing – original draft. **Nursyarizal Bin Mohd Nor:** Supervision. **Perumal Nallagownden:** Supervision, Writing – review & editing. **Irraivan Elamvazuthi:** Visualization. **Mohammad Azad Alam:** Software, Writing – review & editing. **Mohammad Yusuf:** Software, Writing – review & editing. **Mujahid Ali:** Software, Writing – review & editing. **Javed Akhter:** Writing – review & editing. **Mubbashar Mehmood:** Writing – review & editing.

## Declaration of Competing Interest

The authors declare that they have no known competing financial interests or personal relationships which have or could be perceived to have influenced the work reported in this article.
